# Transphyseal Humeral Separations: An Often-Missed Fracture

**DOI:** 10.3390/children10101716

**Published:** 2023-10-23

**Authors:** Arun R. Hariharan, Hans K. Nugraha, Christine A. Ho, Andrea Bauer, Charles T. Mehlman, Paul D. Sponseller, Nathan N. O’Hara, Joshua M. Abzug

**Affiliations:** 1 Paley Orthopedic and Spine Institute, West Palm Beach, FL 33407, USA; 2 Children’s Health Dallas, Texas Scottish Rite Hospital for Children, Dallas, TX 75219, USA; 3 Boston Children’s Hospital, Boston, MA 02115, USA; 4 Cincinnati Children’s Hospital Medical Center, Cincinnati, OH 45229, USA; charles.mehlman@cchmc.org; 5 John Hopkins Children’s Center, Baltimore, MD 21287, USA; 6 Department of Orthopedics, University of Maryland Medical Center, Baltimore, MD 21201, USAjabzug@som.umaryland.edu (J.M.A.)

**Keywords:** transphyseal humeral separation, misdiagnosis, elbow fracture, non-accidental trauma, child abuse

## Abstract

Background: Transphyseal humeral separations (TPHS) are rare injuries often associated with non-accidental trauma, necessitating accurate diagnosis. This study aims to assess the accuracy of diagnosis of TPHS. Methods: A retrospective review was conducted at five academic pediatric institutions to identify all surgically treated TPHS in patients up to 4 years of age over a 25-year period. Demographics, misdiagnosis rates, and reported misdiagnoses were noted. Comparative analyses were performed to analyze the effects of patient age and injury mechanism on misdiagnosis rates. Results: Seventy-nine patients (average age: 17.4 months) were identified, with injury mechanisms including accidental trauma (*n* = 49), non-accidental trauma (*n* = 21), Cesarean-section (*n* = 6), and vaginal delivery (*n* = 3). Neither age nor injury mechanism were significantly associated with diagnostic accuracy in the emergency department (ED)/consulting physician group. ED/consulting physicians achieved an accurate diagnosis 46.7% of the time, while radiologists achieved an accurate diagnosis 26.7% of the time. Diagnostic accuracy did not correlate with Child Protective Services (CPS) involvement or with a delay in surgery of more than 24 h. However, a significant correlation (*p* = 0.03) was observed between injury mechanism and misdiagnosis rates. Conclusion: This multicenter analysis is the largest study assessing TPHS misdiagnosis rates, highlighting the need for raising awareness and considering advanced imaging or orthopedic consultation for accurate diagnosis. This also reminds orthopedic surgeons to always have vigilant assessment in treating pediatric elbow injuries. Level of Evidence: Level III–Retrospective Cohort Study.

## 1. Introduction

Transphyseal humeral separations (TPHS) of the pediatric elbow are relatively uncommon injuries that most often occur in children less than three years of age [1]. Prior to the recent publication of a large series of surgically treated TPHS, very little was known about causes, diagnoses, treatments, or outcomes of these fractures [2].

Knowledge to date has led to the understanding that the fracture occurs in children due to various mechanisms of injury including accidental trauma, non-accidental trauma (NAT), and childbirth [1,3,4,5,6]. Calculating the incidence of this injury has been particularly difficult given the rarity of the injury, the difficulty with identifying missed or non-operatively treated cases, and the additional diagnostic challenge posed by the cartilaginous nature of the elbow in this particularly young population [1,2,7,8,9]. Previous reports have described the use of ultrasound (US) and Magnetic Resonance Imaging (MRI) as useful adjuncts to aid in the diagnosis [10,11]. Additionally, previous reports have shown that surgical treatment has led to good results [2]. Nonetheless, due to the high rates of association with non-accidental trauma, involvement of Child Protective Services (CPS), and presence of additional injuries, accurate and timely diagnosis of this fracture by front-line providers is critical [2,12,13,14,15].

Our prior study has described the epidemiological data and outcome of TPHS [2], yet there is still minimal associated information regarding rates of misdiagnosis. The primary purpose of this study was to assess the rates of misdiagnosis of TPHS amongst radiologists and emergency department (ED)/consulting physicians, as they are often the first line of care for these children.

## 2. Methods

A review was conducted retrospectively at five pediatric institutes to identify every transphyseal humeral separation that was treated surgically in patients aged 48 months or younger from January 1991–December 2016. In addition to Institutional Review Board (IRB) approval obtained from the University of Maryland (IRB# HP-00075350) on 23 June 2017, IRB was also obtained at each of the participating centers. The institutions include the University of Maryland Children’s Hospital, Children’s Health Dallas, Boston Children’s Hospital, Cincinnati Children’s Hospital Medical Center, and Johns Hopkins Children’s Center.

At present, there is no dedicated Current Procedural Terminology (CPT) code for addressing TPHS. Therefore, the CPT code for operative treatment of supracondylar humerus fractures, 24538, was utilized in the database search. A review of the patients’ medical records was performed, including the radiology reports, ED/consulting physician notes, orthopedic operative reports, and radiographs to identify all TPHS patients who had surgery. Patient demographics, mechanism of injury, and Child Protective Services (CPS) involvement were extracted from the medical record. Any additional injuries/fractures were documented.

Using total sampling, any patient with incomplete data was excluded. Consulting physicians were primarily pediatricians caring for the newborn that sustained the injury during the birthing process. This study employed various statistical methods to describe injury epidemiology, diagnostic categories, and accuracies, including the use of frequencies and proportions for categorical data, as well as means with standard deviations (SDs) or medians with ranges for continuous data, depending on data distribution. Additionally, Student’s *t*-tests and chi-squared tests were performed using JMP (Cary, NC, USA) to assess for any association between age and mechanism of injury to accuracy of diagnosis, respectively. The preparation of this manuscript adhered to the Strengthening the Reporting of Observational Studies in Epidemiology (STROBE) guidelines. The study was not supported by external funding.

## 3. Results

Seventy-nine patients aged 0–46 months (mean: 17.6 months), were identified and followed for a median of 57 days post-operatively. The patient demographics are shown in Table 1. The most common mechanism of injury was accidental trauma (*n* = 49), followed by non-accidental trauma (*n* = 21), Cesarean section (*n* = 6), and vaginal delivery (*n* = 3). Child Protective Services were involved in 30 cases (38%).

Additional injuries were reported to involve the appendicular skeleton in seven patients, the axial skeleton in four patients, and the soft tissues in four patients. Some noted additional injuries reported include head injuries, fractures, bruises, burns, and scratches. In terms of imaging, every patient had plain radiographs, 4/79 (5%) had an ultrasound, and 4/79 (5%) had an MRI. Of the seven patients (one patient had both an US and an MRI) who had an US or MRI, none had an inaccurate diagnosis.

Table 2 demonstrates the various fracture misdiagnoses. Some of the most common ones were lateral condyle fractures, supracondylar humerus fractures, dislocations, and medial condyle fractures. There were also numerous diagnoses without classic or typical pediatric elbow fracture diagnoses, such as those listed in the table.

The diagnostic accuracy among radiologists and ED/consulting physicians based on patient age and mechanism of injury are noted in Table 3. The age of the patient had no effect on diagnostic accuracy for radiologists (*p* = 0.17) or ED/consulting physicians (*p* = 0.47). The mechanism of injury did have a statistically significant impact on diagnostic accuracy among radiologists (*p* = 0.03), in that radiologists were more likely to have the correct diagnosis when the mechanism of injury was known. However, this was not the case amongst ED/consulting physicians (*p* = 0.28).

Additionally, the relationships between diagnostic accuracy and delay in surgery as well as involvement of CPS are also displayed in Table 3. For analytical purposes we used diagnostic correctness from just the ED/consulting physician group (*n* = 64), since they are most likely to consult CPS and/or orthopedics for surgical purposes. There was no significant relationship between diagnostic accuracy and delay in surgery (*p* = 1). There was also no significant relationship between diagnostic accuracy and involvement of CPS (*p* = 0.9).

## 4. Discussion

Distal transphyseal humeral separations are very rare injuries that are difficult to diagnose [1,13]. Despite the little information that is available in the current literature regarding this injury, accurate initial diagnosis is critically important due to the association with high rates of non-accidental trauma [2,14,15,16]. The current multicenter study presents injury epidemiology, particularly with regards to rates of misdiagnosis amongst radiologists and ED/consulting physicians, as they are often the first physicians to have any contact with patients with a TPHS. Conducted over an extended duration involving five of the largest pediatric institutions in the United States, this study offers a more comprehensive evaluation of outcomes compared to prior studies. It reveals that both radiologists and ED/consulting physicians exhibit elevated rates of misdiagnosis, with ED/consulting physicians demonstrating slightly better performance.

In newborns and young children, the distal humerus is predominantly composed of cartilage and therefore is particularly vulnerable to elbow injuries [8,13,17]. Nevertheless, in these very young children, clinical findings obtained from physical examination are often limited to non-specific swelling, pain, pseudoparalysis, and/or crepitus about the elbow, making an accurate diagnosis difficult to ascertain based on physical examination alone [1,8,17,18]. Therefore, additional diagnostic modalities and meticulous evaluation are often necessary to arrive at an accurate diagnosis and provide appropriate care to populations with such an injury.

It is important to understand the normal developmental timeline of the elbow with regards to the radiographic appearance on a plain X-ray film. While the sequence of the ossification center’s appearance can exhibit some variation, it is generally observed that the lateral epicondyle, capitellum, and trochlea fuse into a single epiphysis around the age of 13. Subsequently, this epiphysis undergoes fusion with the humeral shaft at approximately 16.5 years of age. In contrast, the medial epicondylar epiphysis, located outside the joint, remains open until the late teenage years. The first ossification center, the capitellum, often does not appear on plain radiographs until between 1 or 2 years of age [18,19]. Following closely, the ossification center of the radial head emerges. It is common to observe dual or multiple ossification centers within the trochlea and olecranon epiphyses. To aid in remembering the sequential order of ossification center appearance in the elbow, the helpful mnemonic “CRITOE” can be employed: capitellum, radial head, internal or medial epicondyle, trochlea, and external or lateral epicondyle [20].

**Figure 1:**
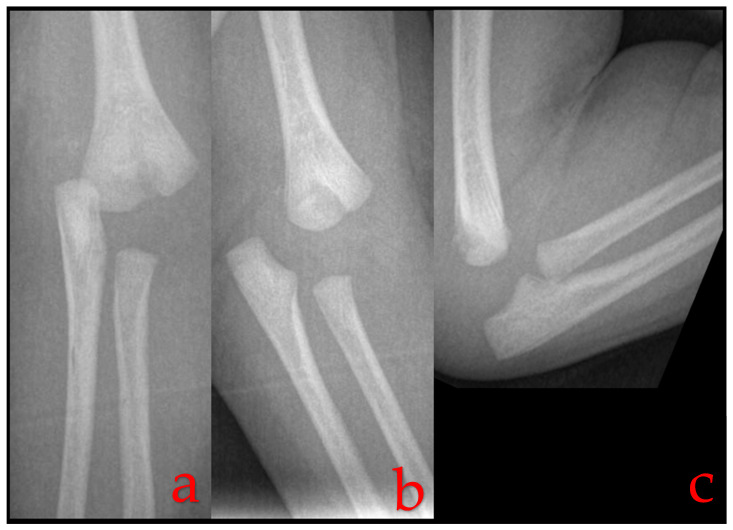
AP, (**a**) oblique (**b**) and lateral (**c**) elbow radiographs of a one-day-old male newborn with an injury attributed to the process of childbirth, revealing a misalignment between the distal humerus and the forearm—a characteristic indication of TPHS.

In TPHS, the most common radiographic finding is posteromedial displacement of the radioulnar complex in relation to the humerus [2,13,21] (Figure 1). This displacement can also take place in other planes and can be a simple Salter–Harris I fracture, or it can be associated with a Thurston–Holland fragment, i.e., a Salter–Harris II fracture [17,22]. Nevertheless, the developmental delay of the ossification center, particularly the capitellum, means that classically used radiographic markers for assessing elbow injuries such as the radiocapitellar line and the anterior humeral line can only be helpful in cases where a capitellar ossification center is present, as these markers rely on the presence and alignment of the capitellum. In children younger than 2 years, these markers may not provide sufficient diagnostic information.

Radiographs of a one-day-old infant’s elbow reveal a misalignment between the distal humerus and the forearm, a characteristic indication of a transphyseal distal humerus separation.

**Figure 2:**
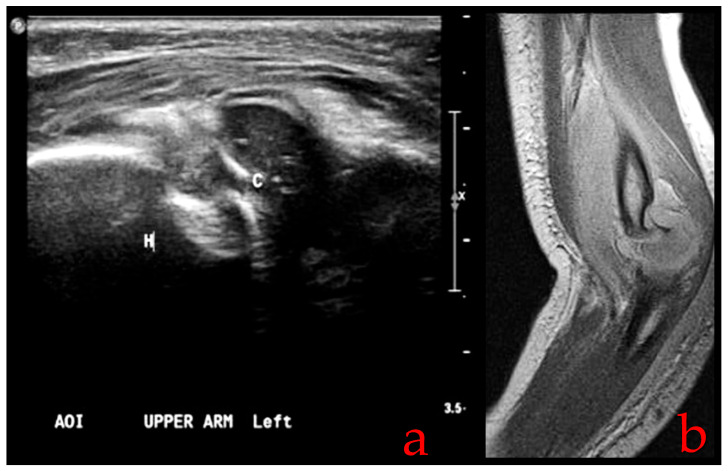
US (**a**) and MRI (**b**) images showing transphyseal distal humerus separation.

To address this challenge, clinicians often turn to alternative diagnostic approaches. One option is to perform comparative radiography, which involves comparing the injured elbow to the unaffected side. This technique can sometimes reveal abnormalities, such as subtle displacements or fractures, by highlighting differences between the two sides. However, in cases where clinical suspicion remains high, but radiographic findings are inconclusive, additional imaging modalities may be necessary. Ultrasound (US) and magnetic resonance imaging (MRI) can aid as valuable diagnostic adjuncts (Figure 2). These modalities offer advantages in terms of providing detailed information about soft tissues, joint alignment, and potential fractures, even in the absence of a visible capitellum on X-rays. An US, in particular, can be a useful tool for evaluating pediatric elbow injuries due to its real-time imaging capabilities. It allows clinicians to assess the joint’s dynamic function and identify abnormalities that may not be evident in static X-rays. While these imaging modalities enhance diagnostic accuracy, they also come with considerations. MRI, for instance, may require sedation for pediatric patients, which introduces its own set of challenges and risks. Additionally, the expertise of the operator is crucial for obtaining high-quality ultrasound images [4,10,11,23,24].

In cases where operative intervention is necessary to manage TPHS or when there is persistent diagnostic uncertainty, intra-operative arthrograms can serve as excellent tools for diagnosis and treatment [1,2,25,26], allowing real-time visualization of the joint’s internal structures and directly guides the surgeon to accurately identify this injury in a timely manner.

This particular study is the most extensive investigation to date to report on the initial evaluation of TPHS. It offers valuable insights into the occurrence, characteristics, and often overlooked aspects of misdiagnosis associated with this injury. TPHS was found to affect children at a relatively early age, typically around 18 months of age. This age bracket is notably younger than the typical age at presentation for other fractures, such as supracondylar and lateral condyle humerus fractures [13].

Another noteworthy finding of this study is the large proportion of non-accidental trauma (NAT) and involvement of Child Protective Services (CPS) in nearly 40% of cases with TPHS. This alarming statistic underscores the paramount importance for accurate and timely diagnosis amongst all healthcare providers involved in the care of these potential victims of abuse [2,14,27].

The data presented in this study build on our existing understanding of TPHS established by our prior work, where we reported on the presentation, diagnosis, and treatment of surgically treated TPHS [2]. Notably, the current study brings to light a previously unreported aspect of TPHS—the notably high rates of misdiagnosis. In particular, it was noted that radiologists, who are an integral part of the diagnostic process, tend to have higher rates of misdiagnosis compared to ED/consulting physicians. One plausible explanation lies in the workflow dynamics between these two groups of healthcare professionals, as ED/consulting physicians often initiate consult with their orthopedic counterpart immediately after identifying a potential fracture, often before completion of their clinical notes. This early engagement in turn would allow for a collaborative discussion between the orthopedic providers and the ED/consulting team and thereby result in a more accurate documentation of the diagnosis. Furthermore, this increased communication would also allow for the orthopedic consultant to educate their colleagues in the ED/consulting team about the intricacies of TPHS.

In contrast, radiologists typically lack direct interactions with both the patients and orthopedic surgeons. They may not engage in discussions with the orthopedic team, nor do they have the opportunity to perform hands-on assessments of patients. This detachment from the clinical context could potentially explain the higher rates of misdiagnosis among radiologists. Their diagnostic interpretations are based solely on radiographic findings, which may not provide the complete clinical picture necessary for accurate diagnosis. Even a small clinical context such as localization cues increased diagnostic confidence and inter-reader agreement, while also reducing the time required for interpreting pediatric fractures on radiographs [28]. Better communication and collaboration between radiologists and orthopedic specialists could therefore potentially enhance the diagnostic accuracy in TPHS cases.

It was also noted that mechanisms of injury had a statistically significant correlation with rates of misdiagnosis amongst the radiologists. Although this finding offers additional insights, it is essential to acknowledge the rarity of TPHS. As such, drawing clinically significant conclusions from this correlation remains challenging. This difference may, perhaps, be due to the existing radiological literature having classically associated TPHS with specific mechanisms of injury, including child abuse and childbirth [4,10,11]. This study has demonstrated that there is a high rate of misdiagnosis among physicians when it comes to TPHS. The fact that this study did not exclusively include board-certified pediatric radiology attendings in the radiologist group could widen its generalizability and clinical ramifications for children in the general pediatric care. Since this study and the majority of previous reports have not discussed missed or non-operatively treated TPHS, the true outcomes of missed or delayed diagnoses and treatments are indeed still rather elusive. Nevertheless, given our understanding that nearly 40% of cases were attributed to NAT, had CPS involvement, or had injuries in addition to the TPHS, it is of paramount importance for any involved physicians other than the orthopedic surgeons to also accurately and in a timely manner identify this injury.

Fortunately, our study yielded some reassuring findings. We observed that there was no significant relationship between accuracy of diagnosis and the timing of surgery. This suggests that, for the cases we examined, delays in surgical intervention did not result from diagnostic inaccuracies. This suggests that there could still be other undocumented factors that play a role as confounding of surgical delay, such as coordination with other specialists or healthcare providers involved in the patient’s care, family preferences and considerations, or insurance and other financial factors.

Another encouraging aspect of our study is the absence of a significant relationship between diagnostic accuracy and involvement of CPS. This suggests that primary healthcare providers are attuned to the atypical nature of these injuries in such young patients. Regardless of the accuracy of the initial diagnosis, the involvement of CPS is not compromised. This underscores the vigilance and commitment of first-line physicians in safeguarding the well-being of these potential victims of abuse.

Several studies have shown a concerning correlation between repeated NAT and an increased rate of mortality, which again underscores the pivotal role played by healthcare providers in recognizing TPHS cases correctly in order to potentially trigger a NAT pathway [29,30]. For example, distinguishing between a triplane and tillaux fracture does not have an impact on the course of care from the perspective of the radiologists or ED/consulting physician. But, distinguishing a supracondylar humerus or lateral condyle fracture from a TPHS may hold substantial clinical implications.

To ensure the best possible care and outcomes for these vulnerable age groups, healthcare institutions should consider implementing a NAT pathway trigger for specific injuries. Currently, many institutions already have a NAT pathway trigger for certain injuries such as femur fractures in children less than 2 years of age. Given the potentially life-altering consequences, a similar trigger should also be considered for TPHS for early identification and intervention, ultimately improving the prospects and safety of any potential victims of abuse.

The rarity of the fracture and the retrospective nature of this study contribute to several limitations inherent in this study. Its retrospective design means that data collection was based only on available documentation and without the ability to cross-reference or validate exhaustive information with the consulting/treating physicians, despite our best effort to eliminate any potential inaccuracies or missing data points. We only looked at patients who were eventually recognized and treated in the operating theater. This could potentially overlook other children with TPHS where it might have been misdiagnosed and thus treated nonoperatively. To mitigate this, and since there is still not a dedicated CPT code for TPHS, and considering that it occurs in the supracondylar region, we employed a broader CPT code for supracondylar humerus fractures in the database search to prevent any potential false negatives. Also, given that only surgically treated patients were able to be identified, the true incidence of TPHS remains elusive. Lastly, despite pooling data across five of the largest institutions in the US over a span of a 25-year period, the study’s sample size remains relatively small, comprising only 79 patients. This limited sample size could potentially underpower some portions of the data analysis. To address these limitations and further enhance our understanding of TPHS, future research endeavors should consider a multi-center design with the use of prospective data to obtain further comprehensive knowledge about these unique injuries.

## 5. Conclusions

Identifying TPHS can certainly be a challenge, even for experienced orthopedic surgeons, due to the fact that it occurs through cartilage and might not be readily seen on the radiographs. It is important to realize that in children under 18 months of age with elbow injuries, commonly known fractures such as supracondylar humerus fractures, lateral condyle fractures, and elbow dislocations are exceedingly rare and are very likely due to TPHS. Therefore, when faced with these findings and the appropriate age group, TPHS should certainly be strongly suggested as part of a viable differential diagnosis, and by maintaining such a high index of suspicion in these specific clinical contexts, the orthopedic team could be involved earlier in the urgent care of these vulnerable patients.

## Figures and Tables

**Table 1:** Patient characteristics.

Variable		N = 79
Age, months, mean (SD)		17.6 (10.4)
Injured side, right, n (%)		50 (50.6)
Mechanism of injury, n (%)		
	Accidental trauma	49 (62.0)
	Non-accidental trauma	21 (26.6)
	C-section	6 (7.6)
	Vaginal birth	3 (3.8)
Additional injury, n (%)		
	Appendicular	7 (8.9)
	Axial	4 (5.1)
	Soft tissue	4 (5.1)
	Both	1 (1.3)
Child Protective Services involvement, yes, n (%)		30 (38.0)

**Table 2:** Common misdiagnoses.

Radiologists	Emergency Department
No Fracture Lateral Condyle Fracture Supracondylar humerus fracture Dislocation Medial Condyle Fracture Metaphyseal fracture of distal humerus Question of boney injury to distal humerus Bucket handle type fracture of distal humerus Comminuted fracture of distal humerus Minimally displaced fracture of distal humerus Oblique fracture of distal humerus Comminuted transcondylar fracture of the distal humerus	Lateral Condyle Fracture Supracondylar humerus fracture Dislocation Medial Condyle Fracture Metaphyseal fracture of distal humerus No fracture Nursemaid’s Elbow Arm fracture Elbow fracture Humerus fracture Distal humerus fracture Salter Harris IV Fracture Arm Swelling

**Table 3:** Patient characteristics and diagnostic accuracy.

		Accurate Diagnosis	Misdiagnosis	*p*-Value
Radiology department		20	55	
Age, month, mean (SD)		14.7 (10.9)	18.6 (10.3)	0.17
Mechanism of injury, n (%)				0.03
	Accidental trauma	11 (55.0)	36 (65.5)	
	Non-accidental trauma	3 (15.0)	16 (29.1)	
	C-section	4 (20.0)	2 (3.6)	
	Vaginal childbirth	2 (10.0)	1 (1.8)	
Emergency department/consulting physician		36	41	
Age, month, mean (SD)		16.8 (11.2)	18.5 (9.8)	0.47
Mechanism of injury, n (%)				0.28
	Accidental trauma	22 (61.1)	26 (63.4)	
	Non-accidental trauma	8 (22.2)	12 (29.3)	
	C-section	5 (13.9)	1 (2.4)	
	Vaginal childbirth	1 (2.8)	2 (4.8)	
CPS Involvement				0.9
	Yes	9	15	
	No	15	25	
Surgical Delay (>24 h)				1.0
	Yes	15	25	
	No	9	15	

## Data Availability

All data and materials support their published claims and comply with field standards and are available for review.
